# Large Outbreak of *Neisseria meningitidis* Serogroup C — Nigeria, December 2016–June 2017

**DOI:** 10.15585/mmwr.mm6649a3

**Published:** 2017-12-15

**Authors:** Chimeremma Nnadi, John Oladejo, Sebastian Yennan, Adesola Ogunleye, Chidinma Agbai, Lawal Bakare, Mohammed Abdulaziz, Amina Mohammed, Mary Stephens, Kyadindi Sumaili, Olivier Ronveaux, Helen Maguire, Debra Karch, Mahmood Dalhat, Martin Antonio, Andre Bita, Ifeanyi Okudo, Patrick Nguku, Ryan Novak, Omotayo Bolu, Faisal Shuaib, Chikwe Ihekweazu

**Affiliations:** ^1^Global Immunization Division, CDC; ^2^Nigeria Centre for Disease Control, Abuja, Nigeria; ^3^Federal Ministry of Health, Abuja, Nigeria; ^4^Africa Centre for Disease Control, Addis Ababa, Ethiopia; ^5^National Primary Health Care Development Agency, Abuja, Nigeria; ^6^Nigeria Country Office, World Health Organization, Abuja, Nigeria; ^7^Nigeria Country Office, United Nations Children’s Fund Abuja, Nigeria; ^8^World Health Organization, Geneva, Switzerland; ^9^Public Health England, London, United Kingdom; ^10^Center for Global Health, Global Rapid Response Team, CDC; ^11^Nigeria Office, Africa Field Epidemiology Network, Kampala, Uganda; ^12^Medical Research Council, Vaccines and Immunity Theme, Banjul, Gambia; ^13^World Health Organization Inter-country Support Team for West Africa, Ouagadougou, Burkina Faso; ^14^Meningitis and Vaccine Preventable Diseases Branch, National Center for Respiratory and Infectious Diseases, CDC; ^15^CDC, Nigeria Country Office, Abuja.

On February 16, 2017, the Ministry of Health in Zamfara State, in northwestern Nigeria, notified the Nigeria Centre for Disease Control (NCDC) of an increased number of suspected cerebrospinal meningitis (meningitis) cases reported from four local government areas (LGAs). Meningitis cases were subsequently also reported from Katsina, Kebbi, Niger, and Sokoto states, all of which share borders with Zamfara State, and from Yobe State in northeastern Nigeria. On April 3, 2017, NCDC activated an Emergency Operations Center (EOC) to coordinate rapid development and implementation of a national meningitis emergency outbreak response plan. After the outbreak was reported, surveillance activities for meningitis cases were enhanced, including retrospective searches for previously unreported cases, implementation of intensified new case finding, and strengthened laboratory confirmation. A total of 14,518 suspected meningitis cases were reported for the period December 13, 2016–June 15, 2017. Among 1,339 cases with laboratory testing, 433 (32%) were positive for bacterial pathogens, including 358 (82.7%) confirmed cases of *Neisseria meningitidis* serogroup C. In response, approximately 2.1 million persons aged 2–29 years were vaccinated with meningococcal serogroup C–containing vaccines in Katsina, Sokoto, Yobe, and Zamfara states during April–May 2017. The outbreak was declared over on June 15, 2017, after high-quality surveillance yielded no evidence of outbreak-linked cases for 2 consecutive weeks. Routine high-quality surveillance, including a strong laboratory system to test specimens from persons with suspected meningitis, is critical to rapidly detect and confirm future outbreaks and inform decisions regarding response vaccination.

## Background

All northern Nigeria states lie within the sub-Saharan “Meningitis Belt,” a region of 26 countries that experiences the largest burden of meningococcal disease, with annual epidemics reported during the December–June dry season. Meningitis causes severe illness, and if not detected and treated quickly, could lead to permanent disability that puts a significant burden on families. In many settings, approximately 10% of meningitis cases ultimately result in death. Before introduction of the meningococcal serogroup A conjugate vaccine (MenAfriVac) in 2013 (*1*), Nigeria experienced some of the largest epidemics of meningococcal meningitis, including the 1996 *N. meningitidis* serogroup A (NmA) epidemic that resulted in 109,580 suspected cases and 11,717 reported deaths (*2*). In 2013, a new strain of *N. meningitidis* serogroup C (NmC) emerged in Nigeria, resulting in small focal outbreaks during 2014–2016 (*3*,*4*). In 2015, this strain of NmC entered neighboring Niger, resulting in the largest ever global epidemic of serogroup C meningitis (*5*), until the 2016–2017 Nigeria epidemic described in this report. Molecular sequencing of bacterial isolates from patients in the region has confirmed the expansion of this new strain of serogroup C in five countries in the region (Ryan Novak, National Center for Immunization and Respiratory Diseases, CDC, personal communication, 2017).

## Case Definition and Incidence Thresholds for Response

A suspected case of meningitis was defined as the sudden onset of fever (>100.4°F [>38.0°C]) and at least one meningeal sign, including neck stiffness or altered consciousness in any person, or a bulging anterior fontanelle in children aged <18 months (*6*). Available cerebrospinal fluid (CSF) or blood specimens from patients meeting the suspected meningitis case definition were transported to a designated laboratory for confirmation by culture, latex agglutination, or real-time–polymerase chain reaction (PCR) tests. World Health Organization (WHO) Meningitis Outbreak Response Guidelines were used to identify geographic areas at risk for epidemics to guide response (*6*). Attack rates of suspected meningitis cases reported weekly by LGAs were calculated. WHO recommends that a set of preparedness activities be implemented when the attack rate of suspected meningitis in an LGA crosses a defined “Alert” threshold, and additional response activities at a defined “Epidemic” threshold (Table 1).

**TABLE 1:** Guidelines for incidence thresholds and interventions for detection and control of epidemic meningococcal meningitis based on population size of the local government area in countries in Africa with endemic disease* — World Health Organization

Incidence threshold	Population size		Interventions
	<30,000	30,000–100,000	
**Alert**	Two suspected cases in 1 week or increase in incidence compared with nonepidemic years	Three suspected cases per 100,000 population per week (two or more cases in 1 week)	1) Inform authorities, 2) strengthen surveillance, 3) investigate, 4) confirm (including laboratory), 5) treat cases, 6) prepare for eventual response
**Epidemic**	Five suspected cases in 1 week^†^ or doubling of number of cases in a 3-week period	10 suspected cases per 100,000 population per week	1) Conduct mass vaccination^§^ within 4 weeks of crossing epidemic threshold, 2) distribute treatment to health centers, 3) treat according to epidemic protocol, 4) inform the public

* Guidelines adapted from http://apps.who.int/iris/handle/10665/144727.

^†^ In special situations such as mass gatherings, refugees, displaced persons or closed institutions, two confirmed cases in a week should prompt mass vaccination.

^§^ If an area neighboring one targeted for vaccination is considered to be at risk (e.g., cases early in the dry season, no recent relevant vaccination campaign, or high population density), it should be included in a vaccination program.

## Outbreak Investigation

Two outbreak investigation teams were deployed to Zamfara and Sokoto states to augment routine surveillance, forward available CSF specimens to a designated laboratory for analysis, verify the extent of the outbreak, and gather specific information regarding the affected population to guide response. The first meningitis cases, a 21-case cluster in a village in Zurmi LGA of Zamfara State, were reported to the State Ministry of Health in December 2016; however, the cluster was not reported to NCDC until February 2017, after the outbreak had spread to four other LGAs in Zamfara, and to Katsina, Kebbi, Niger, and Sokoto states. During December 2016–June 2017, among Nigeria’s 37 state-level jurisdictions, 26 (70%) reported suspected meningitis cases, with peak incidence during reporting week 15 (April 16–22, 2017) (Figure). Meningitis incidence in 56 LGAs met the alert threshold and in 38 met the epidemic threshold. Overall, 14,518 suspected cases and 1,166 deaths (case-fatality ratio = 8.0%), were reported during the outbreak; 7,140 (49%) cases were reported from Zamfara State, and 6,792 (47%) occurred in children aged 5–14 years (Table 2). Confirmatory laboratory testing was conducted for specimens from 1,339 (9%) suspected meningitis patients; among these, 433 (32.3%) were laboratory-confirmed as bacterial meningitis, including 358 (82.7%) with NmC (Table 2).

**FIGURE:**
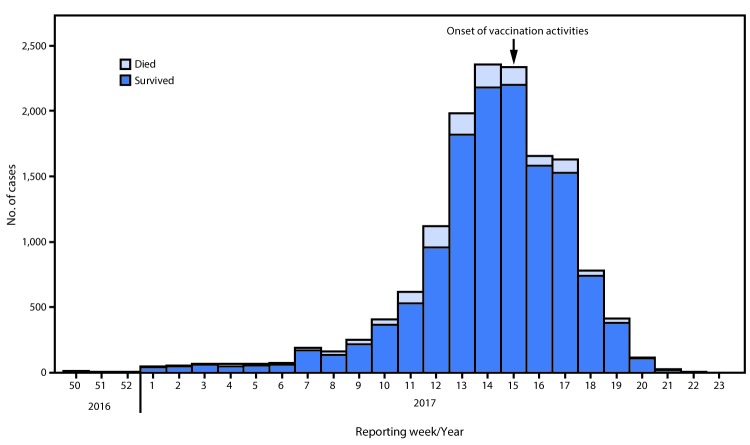
Weekly number of suspected meningitis cases — Nigeria, December 2016–June 2017* * Reporting week 15 corresponds to April 16–22, 2017; week 21 corresponds to June 4–10, 2017.

**TABLE 2:** Characteristics of patients in 14,518 suspected cerebrospinal meningitis cases — Nigeria, December 2016–June 2017

Characteristic	No. (%)
**Sex**	
Male	7,802 (53.7)
Female	6,699 (46.2)
Missing/Unknown	17 (0.1)
**Age group (yrs)**	
<1	219 (1.5)
1–4	1,796 (12.4)
5–14	6,792 (46.8)
≥15	5,667 (39.1)
Missing/Unknown	44 (0.3)
**State**	
Zamfara	7,140 (49.2)
Sokoto	4,980 (34.3)
Katsina	915 (6.3)
Yobe	415 (2.9)
Kebbi	142 (1.0)
Niger	131 (0.9)
Other	795 (5.5)
Meningococcal serogroup or other identified organism*^,†^	
A	27 (6.2)
B	1 (0.2)
C	358 (82.7)
W	1 (0.2)
X^§^	—
Y	0 (0)
Unknown	32 (7.4)
*Haemophilus influenzae *(type b)	5 (1.2)
*Streptococcus pneumoniae*	9 (2.1)

*Total number of laboratory specimens tested = 1,339; 433 specimens yielded meningococcal or nonmeningococcal organisms. A total of 129 test results were invalid or missing, and the rest were classified as negative for any organisms tested.

^†^ Cases confirmed by any of the following tests: latex agglutination, polymerase chain reaction, or culture.

^§^ Laboratory tests not available to detect *Neisseria meningitidis* serogroup X.

## Early Outbreak Response Activities

Following initial investigations, including health facility register reviews and analysis of community informant reports, NCDC activated the meningitis EOC on April 3, 2017 to coordinate outbreak response strategies and operations across the entire country in collaboration with country partner agencies, including WHO, CDC, the Africa Centre for Disease Control and Prevention, the United Nations Children’s Fund (UNICEF) and the Africa Field Epidemiology Network. To ensure that suspected meningitis cases were rapidly detected and investigated, meningitis surveillance, according to WHO’s Africa Region Guidelines for Enhanced Meningitis Surveillance, was strengthened in all states, regardless of whether states reported cases. EOCs were also activated to coordinate outbreak response activities in Sokoto and Zamfara states, the two states at the epicenter of the outbreak. Rapid response teams of epidemiologists and clinicians were deployed from the national EOC to support states with at least one LGA meeting the defined outbreak threshold.

Early outbreak response activities were hampered by difficulty in accessing some of the more rural and remote communities experiencing the outbreak. A limited capacity for CSF specimen collection among health care workers, deficiencies in the laboratory systems, including a lack of basic test kits and limited resources to support timely and appropriate specimen transportation from health facilities to a laboratory with PCR or culture capacity, contributed to delayed case identification. Additionally, the human resources needed to support effective outbreak detection and response were limited in some of the states with the largest case numbers, necessitating the recruitment and deployment of a large contingent of ad hoc technical support personnel from the national level to support outbreak control activities in these states.

## Outbreak Response Vaccination

The National Primary Health Care Development Agency, responsible for vaccination activities in Nigeria, received meningococcal C–containing vaccines through the International Coordinating Group on Vaccine Provision in April 2017, 2 months after the outbreak was first widely reported. Because of limited vaccine supplies, vaccine use was prioritized to the most affected LGAs in Katsina, Sokoto, Yobe, and Zamfara states (*6*) where approximately 2.1 million (84.4%) of an estimated 2.5 million persons at risk (based on the WHO guidelines) aged 2–29 years were vaccinated. Extensive social mobilization activities, including outreach to community leaders and engagement on social and traditional media helped raise awareness and facilitate desired behavior change, including vaccine acceptance and avoidance of overcrowding, thereby reducing potential for continued transmission.

## Discussion

The outbreak likely represents the largest global outbreak of NmC. Response measures implemented during the outbreak, including improved case finding and management as well as mass vaccination campaigns, might have contributed to the outbreak control. However, the large number of cases and prolonged duration of the outbreak highlight key lessons for meningitis outbreak prevention, detection, and response in Nigeria and other countries in the meningitis belt. Timely and appropriate use of meningococcal vaccines is effective in preventing and limiting the spread of meningococcal meningitis outbreaks. The introduction of the meningococcal A conjugate vaccine against NmA in Nigeria and other countries in the meningitis belt represents a major milestone in meningitis outbreak control and has contributed to significant reductions in NmA infections (*7*,*8*). However, laboratory data from this and other recent outbreaks point to the evolving regional meningitis epidemiology with increasing proportions of epidemics attributable to bacterial meningitis pathogens other than NmA, for which meningococcal A conjugate vaccine provides no protection (*3*,*4*). These findings suggest an urgent need to expand availability of multivalent vaccines that are effective against non-A serogroups.

In Nigeria, meningitis is classified as an epidemic-prone disease, requiring immediate notification, investigation, and necessary action (*9*); significant lapses in reporting in the early stages of this outbreak (from December 2016 to February 2017) might have contributed to its large size and wide reach. Additionally, limited capacity for CSF specimen collection, a lack of test kits, and inadequate resources to support timely and appropriate specimen transportation from health facilities to a laboratory with PCR or culture capacity contributed to the low percentage of confirmed meningitis cases. Similarly, delays in case finding, reporting and investigation, especially in the more remote areas, limited timely outbreak response. These meningitis surveillance system weaknesses merit further investigation, with remediating action implemented to prevent future reoccurrence. Because delayed access to meningococcal vaccines might have contributed to the prolonged outbreak duration, a careful examination of country vaccine requisition processes, and International Coordinating Group on Vaccine Provision protocols for vaccine requests, approval, delivery and use, is needed.

A surveillance and outbreak response system is most effective when the capacity to prevent, detect, and appropriately respond to outbreaks is available (*10*). In Nigeria, the human resource capacity to support an effective outbreak response varied widely within and between states, and was severely limited in some of the most at-risk states and LGAs. In low human resource capacity settings, evolving and refining new models for effective and timely outbreak detection and response, including scaling up emergency Rapid Response Team deployment where needed, is critical. In Nigeria, an opportunity exists for improved response coordination with lessons learned from EOCs established for coordination of polio eradication activities and response to Ebola virus disease, as well as leveraging trained personnel from the Nigeria Field Epidemiology and Laboratory Training Program. In the longer term, building adequate health care worker capacity at all national and subnational surveillance system levels will be essential to a timely and effective outbreak response. Functional laboratory systems are pivotal to meningitis case confirmation and provide guidance for critical outbreak response activities, including decisions on appropriate vaccine use.

With the outbreak now declared over, efforts to improve surveillance and outbreak preparedness for meningitis need to continue. Recently concluded national and regional evaluations of the outbreak response have articulated recommendations for improving meningitis outbreak prevention, timely detection, and response in Nigeria, and implementation of these recommendations is needed at all levels of the public health system. Additionally, conducting a review of the implementation of current meningitis outbreak alert and epidemic thresholds in Nigeria, including an assessment of sub-LGA–level sensitivity to outbreaks at the current thresholds could help to ensure optimal and timely detection at the lower levels. Developing and introducing conjugate vaccines effective against non-A meningococcal serogroups might help reduce the risk for future non-serogroup A meningococcal meningitis outbreaks.

**Summary**

**What is already known about this topic?**

Meningococcal disease caused by *Neisseria meningitidis* causes severe illness, and could lead to permanent disability or death if not quickly detected and treated. The largest global burden of meningococcal disease is in sub-Saharan Africa, where annual epidemics caused mainly by *N. meningitidis* serogroup A were previously common. After the introduction of meningococcal A vaccines in 2013, meningitis caused by serogroup A declined. However, *N. meningitidis* serogroup C (NmC) has now emerged as a cause of large outbreaks.

**What is added by this report?**

During December 2016–June 2017, the largest global epidemic of meningitis caused by NmC occurred in northern Nigeria, with 14,518 suspected cases and 1,166 deaths reported. An emergency operations center coordinated rapid development and implementation of an emergency outbreak response plan, including administration of meningococcal serogroup C–containing vaccines to >2 million persons. Multiple logistical challenges were encountered during the response; the outbreak was declared over in June 2017.

**What are the implications for public health practice?**

National and regional evaluations of the outbreak response have outlined recommendations for improving meningitis outbreak prevention, timely detection, and response in Nigeria. Implementation of these recommendations will be key to reducing future meningitis outbreaks. Expanding availability of multivalent vaccines that are effective against non-A serogroups of *N. meningitidis* might prevent future outbreaks in this region.
